# ZNStress: a high-throughput drug screening protocol for identification of compounds modulating neuronal stress in the transgenic mutant sod1G93R zebrafish model of amyotrophic lateral sclerosis

**DOI:** 10.1186/s13024-016-0122-3

**Published:** 2016-07-26

**Authors:** Alexander McGown, Dame Pamela J. Shaw, Tennore Ramesh

**Affiliations:** Sheffield Institute for Translational Neuroscience (SITraN), University of Sheffield, 385A Glossop Road, Sheffield, UK

## Abstract

**Background:**

Amyotrophic lateral sclerosis (ALS) is a lethal neurodegenerative disease with death on average within 2–3 years of symptom onset. Mutations in superoxide dismutase 1 (SOD1) have been identified to cause ALS. Riluzole, the only neuroprotective drug for ALS provides life extension of only 3 months on average. Thishighlights the need for compound screening in disease models to identify new neuroprotective therapies for this disease. Zebrafish is an emerging model system that is well suited for the study of diseasepathophysiology and also for high throughput (HT) drug screening. The mutant sod1 zebrafish model of ALS mimics the hallmark features of ALS. Using a fluorescence based readout of neuronal stress, we developed a high throughput (HT) screen to identify neuroprotective compounds.

**Results:**

Here we show that the zebrafish screen is a robust system that can be used to rapidly screen thousands ofcompounds and also demonstrate that riluzole is capable of reducing neuronal stress in this model system. The screen shows optimal quality control, maintaining a high sensitivity and specificity withoutcompromising throughput. Most importantly, we demonstrate that many compounds previously failed in human clinical trials, showed no stress reducing activity in the zebrafish assay.

**Conclusion:**

We conclude that HT drug screening using a mutant sod1 zebrafish is a reliable model system which supplemented with secondary assays would be useful in identifying drugs with potential for neuroprotective efficacy in ALS.

**Electronic supplementary material:**

The online version of this article (doi:10.1186/s13024-016-0122-3) contains supplementary material, which is available to authorized users.

## Background

Amyotrophic lateral sclerosis (ALS) is a progressive neurodegenerative disorder that leads to death on average within 2–3 years of symptom onset. It is characterised by the progressive loss of upper and lower motor neurons in the motor cortex, brainstem and spinal cord leading to muscle wasting, weakness and eventual paralysis. ALS is predominantly a sporadic disease, but 5–10 % of cases are familial, usually with autosomal dominant inheritance. Over 150 mutations in superoxide dismutase 1 (SOD1) have been identified to cause ALS and several of these mutations have been modelled in multiple species, including mice and zebrafish [1–5]. The only drug currently approved for slowing disease progression in ALS is riluzole, which gives ALS patients a life extension of only 3 months on average [6]. This highlights the need for compound screening in disease models to identify new neuroprotective therapies for this devastating human disease.

High throughput screening (HTS) assays underpin drug discovery efforts as they enable rapid screening of a large library of bioactive molecules in multiple disease models. High throughput screens are typically classified as either target directed drug discovery screens (TDDS) or as phenotypic drug discovery screens (PDDS) [7, 8]. In target based screens manipulation of a known molecular target is the primary goal, with a main focus on the use of technology for generating throughput. In contrast, phenotypic screens use a top—down approach, where a disease process is manipulated in a screen and the assay uncovers compounds that directly impact on the disease process. Phenotypic screens typically have a lower throughput due to the complexity of the pathways and models used [7, 8]. With advances in genomics and the identification of molecular targets for many diseases, target-based approaches have been the main drivers of drug discovery in the 20^th^ and 21^st^ centuries. However, a recent report indicates that phenotypic screens are still the main providers of new-in-class drugs emerging into the clinic [9]. Among the 45 first-in-class drugs approved by the FDA during the period of 1999 to 2008, 28 drugs were discovered using PDDS, while only 17 were discovered by applying the TDDS method [9]. This occurred despite the fact that the majority of drug discovery efforts during this period were primarily geared towards TDDS based approaches [9].

Neurodegeneration is a field where target based approaches are yet to convincingly demonstrate utility in drug discovery. Furthermore, drug failure rates from bench to clinic in the CNS arena are far higher than in any other disease areas [10]. The lack of understanding in the molecular mechanisms that underlie neurodegeneration, and the lack of clear and specific targets, have played an important role in the poor success rates of drug discovery in neurodegeneration [11]. This highlights a need for new models and carefully designed screens/trials within the neurodegenerative field. The advantages of using PDDS in neurodegeneration are particularly compelling, as the assays developed will be generally unbiased towards a specific target, and may be able to modulate a functional phenotype associated with disease aetiology, multiple molecular pathways and/or symptoms in human patients.

Zebrafish (*Danio rerio*) are now widely used as in vivo models for many human neurological diseases, including ALS [2, 3, 5, 12–14]. Zebrafish are very useful for modelling human disease and drug screening due to their rapid development, large numbers of offspring, external fertilisation, small size, susceptibility to genetic manipulation and transparency during development, making them an excellent model system for imaging [3, 5]. Zebrafish are an increasingly powerful model in the field of high-throughput in vivo compound screening and have been used to assess both drug efficacy and toxicity [15, 16]. The development of a zebrafish Sod1 G93R model of ALS showed that zebrafish mimic many aspects of the human disease [5]. The mutant Sod1 zebrafish showed hallmark features of ALS that included impaired swimming ability, reduced muscle strength, neuromuscular denervation and loss of motor neurons [5]. Additionally, we have recently shown that inhibitory interneurons are primarily affected at the embryonic stage in this zebrafish model, long before the motor neurons exhibit pathological changes [3]. This suggests that protection of the inhibitory interneurons at this early embryonic stage may delay the motor neuron degeneration observed in adult zebrafish. These findings are in keeping with recent evidence from human patients indicating that ALS is a disease with a prolonged prodromal stage which may warrant early intervention [17–22].

Heat shock proteins (HSP’s) are ubiquitously expressed and found in all organisms. These proteins are up-regulated in response to increased temperature as well as other forms of stress, including cellular stress [23, 24]. Heat shock proteins were first identified in 1962 in a *Drosophila* model where an increase in temperature was seen to induce new RNA synthesis [25]. The main function of the heat shock response is as a protective mechanism involved in the unfolded protein response (UPR), to overcome and promote cell survival in the face of a toxic insult, such as the presence of mutant or misfolded protein species, as is the case in SOD1 mutations. The heat shock response induces the synthesis of a family of heat shock proteins, including hsp70, that act as chaperones, which attempt to refold misfolded proteins or target them for degradation. Therefore heat shock proteins represent an excellent marker of cellular stress and can be used as a readout for mutation driven toxicity. In fact western blotting analysis in the sod1 G93A mouse model of ALS, demonstrated up-regulation of Hsp70 in the brain and spinal cord at 8 months of age [26].

**Fig. 1:**
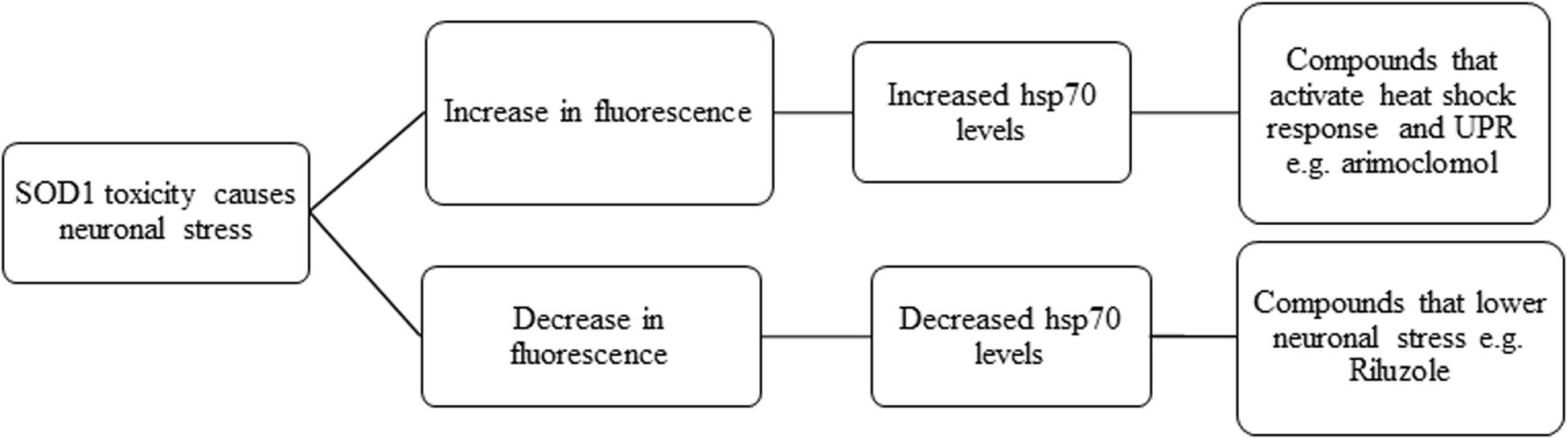
Flow chart of the screening outcomes. The ZF assay can be used to identify neuroprotective compounds that act at different points in the cascade of mutant sod1 mediated toxicity. Inhibitors would potentially impact the earliest event in sod1 toxicity by reducing the toxic effects of mutant sod1. Activators may potentially further activate the neuroprotective stress response thus ameliorating the effects of sod1 toxicity

Using an Hsp70-DsRed reporter gene we have developed a neuronal stress readout in a mutant Sod1 zebrafish model of ALS [5]. As the inhibitory interneurons are affected at the early embryonic/larval stages and exhibit activation of a heatshock stress response, we deduced that a readout of hsp70 activation by quantitation of the DsRed fluorescence signal could generate a simple high throughput screen suitable for the identification of modulators of neuronal stress in the zebrafish model. Interestingly, we found that riluzole, the only neuroprotective therapy authorised for the treatment of ALS, was able to reduce the neuronal stress signalled by hsp70 activation. We sought to develop an in vivo high throughput screen based on the identification of compounds which could modulate the quantifiable fluorescent readout of neuronal stress. Our screen utilises a high-capacity liquid handling system and a high-content imaging system to deliver high-throughput drug screening in an in vivo model. By screening for compounds that activate or inhibit the hsp70-DsRed response, we can identify compounds that act upstream or downstream of the mutant Sod1 toxicity pathway (Fig. 1). Thus, this hybrid assay combines a target based approach that can differentiate upstream and downstream pathways and at the same time has the advantages of a phenotypic screen, as it involves measuring the pharmacological modulation of a pathophysiological readout. In this study we describe the identification, optimisation, quality control and statistical analysis of the zebrafish neuronal stress (ZNStress) assay using a mutant *Sod1 G93R* zebrafish model of ALS. The Spectrum library of 2000 compounds was screened in the ZNStress assay, to identify modulators of neuronal stress using an *hsp70*-*DsRed* readout as a marker of neuronal stress. We demonstrate here the reproducibility and throughput of the assay and the ability of a phenotypic in vivo screen to match the statistical robustness of a TDDS.

## Methods

### Animals

Adult and larvae zebrafish (*Danio Rerio*) were kept at the University of Sheffield Zebrafish Facility, maintained at 28.5 °C and bred according to established procedures [27]. Animal protocols were undertaken in line with a Home Office approved project licence. The care and maintenance of animals were performed under the Home Office project licence as per ASPA regulations. All experiments were performed with embryos generated by out crossing the G93Ros10-SH1 line with the wildtype AB zebrafish strain [3].

### Compound library and storage conditions

The spectrum library (Microsource Inc) is a collection of 2000 compounds from the US drug collection, international drug collection and natural plant compounds. The Library is stored in deep well storage plates within the SPOD system (Roylan) to prevent library deterioration. The SPOD system is a specialised drug storage system designed to extend the lifespan of compound libraries by controlling environmental conditions (atmospheric pressure of 0.5PSI, oxygen level <10 %, relative humidity <5 %) and maintaining an inert environment by mixing with N2. To create a screening library, the stock library was imprinted onto 384 well LDV plates (Labcyte) before dilution to 10 mM to generate a final well volume of 10 μM. All drugs used in the ZNstress assay and spectrum library were solubilized and delivered in DMSO.

### ZNStress assay protocol

Embryos were manually dechorinated at 24 h before loading into 96 well plates (Grenier BioOne, μClear) in 70 μl of E3 media and imaged using the Incell Analyser 2000, high-content imaging system (GE healthcare) to genotype the zebrafish prior to initiating the screen for DsRed fluorescence. Only normally developed healthy appearing embryos with expected fluorescence patterns are selected for screening (Additional file 1: Figure S1). All drugs were solubilized in DMSO with a final maximal DMSO concentration of 0.1 % and hence 0.1 % DMSO is used as a negative control. The drugs were loaded onto screening plates using the Echo 550 liquid handling system (Labcyte) to accurately and rapidly transfer the spectrum library of compounds into assay plates at a final drug concentration of 10 μM in 200 μl. The zebrafish transfered into the drug solution at 48hpf and incubated at 28 °C until 6dpf. The plates are monitored each day for death and imaged again at 6 dpf using Incell Analyzer. Wells with dead and defective embryos are noted and excluded. The zebrafish larvae are then terminally anaesthetised using MS-222 (Sigma), transferred individually in 50 μl to V-bottom 96 well plates (Grenier, Bio-One) and each well is then sonicated for 5 s at 25 % amplitude before being centrifuged at 3000 rpm for 15 min. 20 μl of supernatant is then loaded into 384 well plates and the fluorescence measured using the OMEGAstar plate reader system for emission in the DsRed wavelength (BMG Labtech).

### In cell and Pherastar imaging

For automated imaging the InCell Analyzer 2000 (GE Healthcare) was used to image embryos before using the zebrafish segmentation plugin (GE Healthcare) which allows the user to analyse the fluorescence in individual tissues. The software segments the zebrafish based upon the body shape into individual areas such as eye, brain, liver and spinal cord. Unfortunately, the software currently only segments the zebrafish accurately at 4dpf and this limitation introduced variability, making the use of this software unsuitable for the assay. Fluorescence well scanning was performed using the Pherastar FS system (BMG Labtech, 15x15 well scan). This method allows the user to keep the zebrafish alive after the assay. Unfortunately, the well scanning assay is slow for large assays (3–4 h per 96 well plate) and thus drastically reduced the maximum throughput of the assay.

### Data analysis and quality control for ZNStress assay

Strictly Standardised Median Difference (SSMD) was used as the criterion for hit selection in the ZNStress assay [28]. In HTS screening an important quality control is to look at how much the positive controls, negative controls and tested compounds differ from one another. Readouts such as the Z-Score, signal to noise (S/N) and signal to background (S/B) are commonly used in HTS as readouts. These readouts work by comparing the values of two different well types in an assay. S/N and S/B only take the variability of one group into account and cannot provide quality control data on an assay with multiple groups. The Z-score takes into account the variability of two groups being analysed but not of the whole plate and so does not control the false positive/negative rate accurately. In addition, these analysis methods do not take into account the strength of difference between a test compound and a negative reference directly, but as a mean value, meaning that weaker hits can be missed. The SSMD calculates the median of differences divided by the standard deviation of the differences between a test compound and a negative reference [29] and therefore represents the average fold change penalised by the variability of the fold change across the plate. SSMD is a more suitable screen readout for in vivo HTS screening as it takes into account the variability across the whole plate, giving each well a readout shown as the magnitude of the difference in fluorescence compared to the whole plate, resulting in a more meaningful biological association. For this assay a threshold of *B* value < -0.5 or >0.5 was taken as the cut-off value to identify hit compounds for further investigation (Additional file 2: Table S1).

## Results

### Feasibility of Pherastar assay for high throughput fluorescence quantitation

Pherastar is a fluorescence plate reader that can measure fluorescence in a matrix format. This allows quantitation of fluorescence within each well, with high resolution at hundreds of points. We established that, as opposed to an image based quantification system (InCell), a direct high resolution fluorescence detector would be better for quantitating the DsRed fluorescence while still maintaining a degree of anatomical specificity. The Pherastar readout in Additional file 3: Figure S2 demonstrates the reduction in DsRed signalling following exposure to riluzole. The top rows show riluzole treated zebrafish, while the bottom row shows the control zebrafish exposed to DMSO. The data show a large decrease in hsp70-DsRed activation in the riluzole treated zebrafish, particularly in the head/brain region. However, consistent orientation of embryos created a problem in obtaining reproducible results. Also, the system was relatively slow and therefore impacted on the screen throughput capabilities. Hence, although this assay was more sensitive than InCell imaging, as it provided a signal intensity based readout, it was not capable of handling large throughput screening in a reliable fashion.

### Use of whole embryonic extract for high throughput fluorescence quantitation

**Fig. 2:**
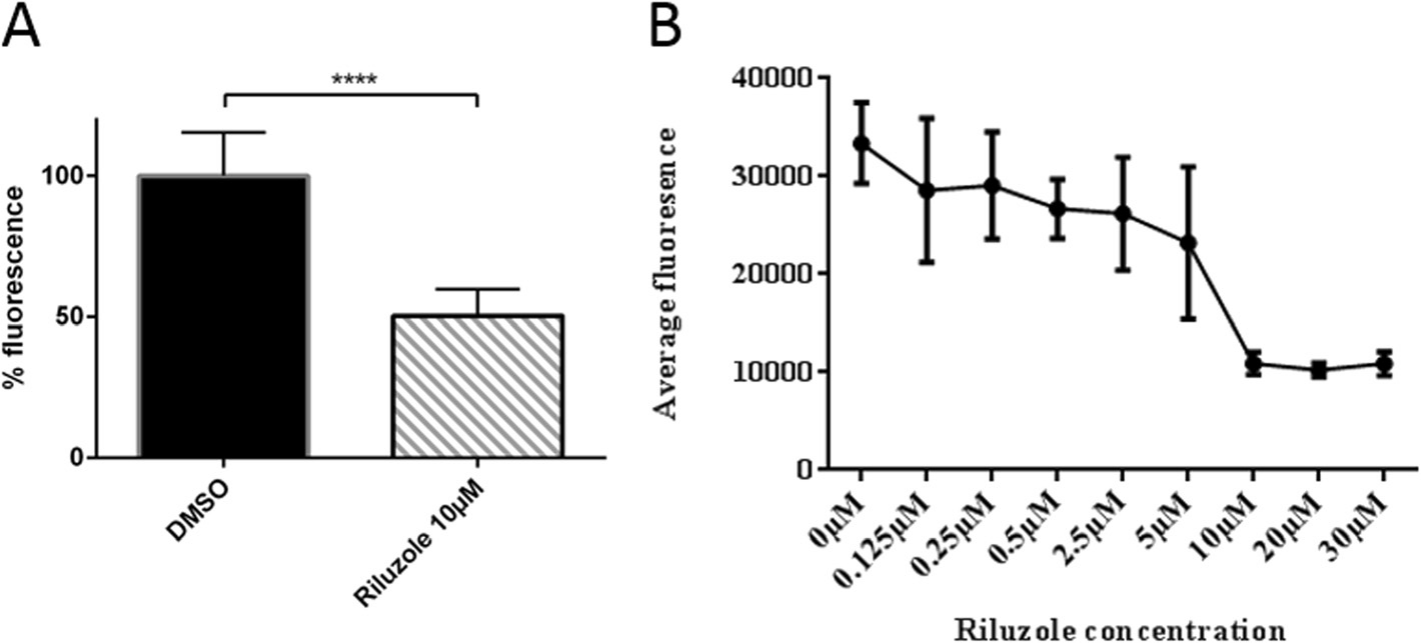
Treatment with riluzole leads to significant quantifiable reduction in neuronal stress. **a** Zebrafish treated with riluzole show a large reduction in DsRed fluorescence after treatment with riluzole at 10 μM. *N =* 19 fish per condition, error bars show SD. *P <*0.0001. **b** Dose response analysis was performed in 96 well plates with 1 embryo/well immersed in varying doses of riluzole (0-30 μM). *N =* 12 fish per concentration. Error bars show SD

While InCell imaging and the Pherastar system provide an excellent way to measure neuronal stress levels, both of these systems require careful positioning of embryos, sophisticated image analysis software and computation time to analyse the results. We observed that riluzole reduced cellular stress in the CNS, and that the CNS was the primary source of DsRed fluorescence. We decided to determine if whole embryo extracts would be sufficient to measure the change in fluorescence levels. Such an assay, if shown to be robust would be beneficial as a high throughput readout, with enhanced reproducibility and reduced variability. Towards this we homogenised the zebrafish larvae by sonication and measured the fluorescence of the lysate, to determine whether robust inhibition of DsRed fluorescence was observed in the presence of the positive control compound, riluzole. Treatment of embryos from 2dpf to 6dpf with 10 μM riluzole reduced the DsRed fluorescence by approximately 50 % (Fig. 2a).

One important aspect of a screen is the success of identified hit compounds to show a concentration dependent response in the subsequent validation stage. To determine if the embryo extracts could be utilized to measure a dose response to riluzole, embryos were exposed to increasing doses of riluzole and the fluorescence measured. Fluorescence quantitation showed a clear dose-dependent reduction in fluorescence (Fig. 2b), thus confirming the usefulness of this assay in providing a good quantitative method for high throughput screening and drug effect.

### Reduced between and within plate variability when assessing positive and negative controls using embryonic extract

As the first step prior to increasing throughput, we determined the assay-to-assay variability. The only source of variation observed is the day to day variation, due to the differences in the animals used. To reduce this we measured the variability observed with the positive and negative control compounds, between and within plates (Additional file 4: Figure S3). This variability was found to be minimal, suggesting that biological variation between animals should not greatly impact upon the success of the assay. The quality control analysis of the control compounds within the plate highlighted the applicability of this model to identify compounds such as riluzole that reduce the fluorescence readout of neuronal stress.

### Development and optimisation of the high-throughput screen

Optimisation of a high-throughput screen was focused on increasing the number of compounds screened per week without compromising the sensitivity or specificity of the assay. Multiple screen lengths were investigated. The screen was begun at 2dpf as this allows genotyping before the assay begins, reducing the number of compounds per zebrafish, as well as allowing the zebrafish to have a further developed nervous system, compared to 24hpf. Shorter assays of 2 and 3 days of drug exposure showed drug effect, but the reduction in fluorescence was smaller, which reduces the effect window available for hit detection. This led to an increased incidence of false positives and negatives in quality control (QC) experiments. Drug exposure for 4 days gave a large window of drug effect, so that compounds only having a subtle effect were still detectable, which was not be possible with the shorter drug exposure times.

**Fig. 3:**
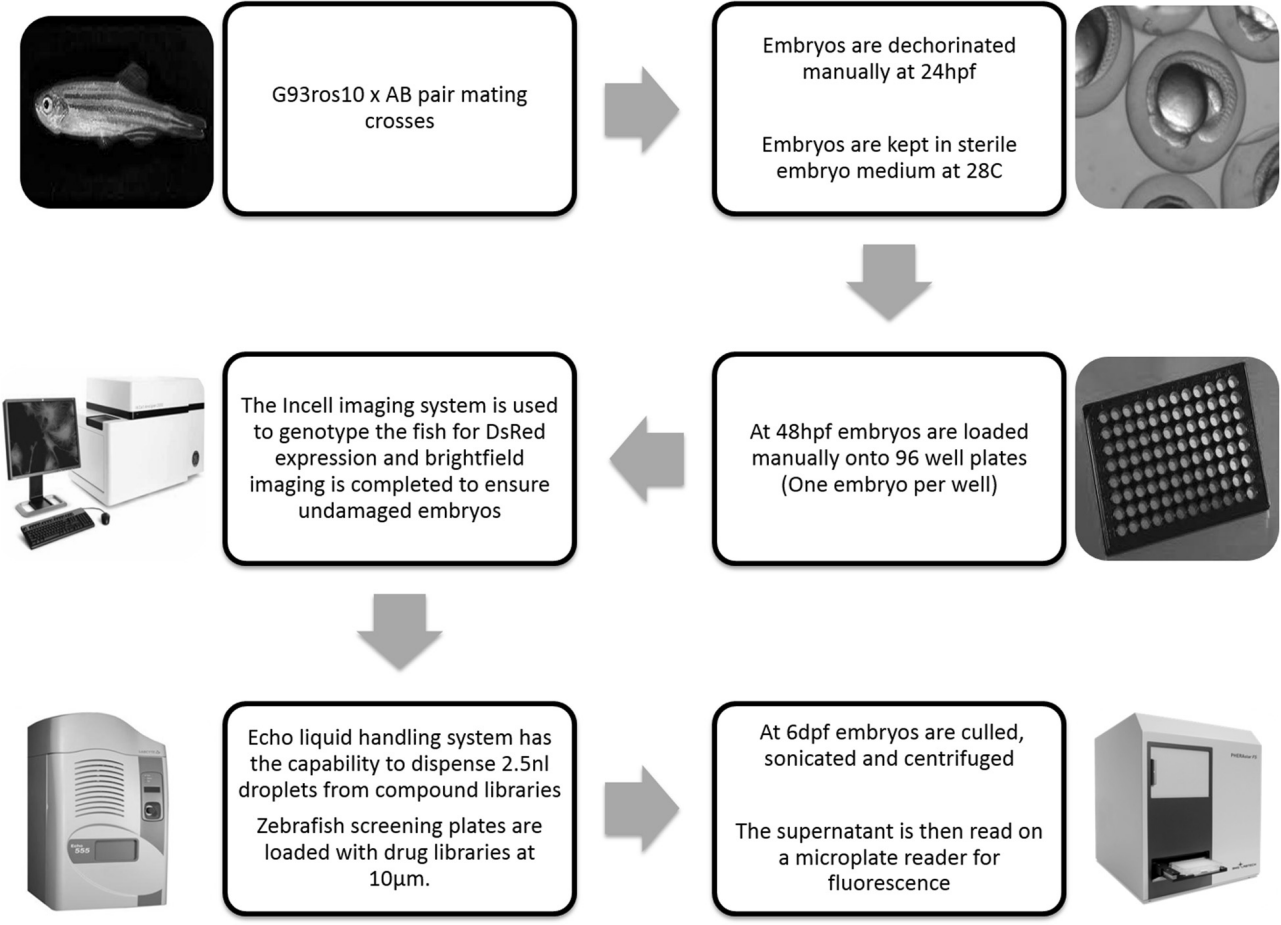
Screening flowchart showing the systems used to generate the high-throughput screen

Multiple endpoint readouts were investigated to identify the readout that reproducibly gave the most accurate results while maintaining a high-throughput. Implementation of robotics systems such as the Echo 550 liquid handling system (Labcyte) and the InCell 2000 (GE healthcare) Analyzer further improved the accuracy and throughput of the screen by automating the genotyping and dosing steps. The schematic of the final optimised screening process is shown in Fig. 3.

### The high-throughput screen

#### Development of HT assay QC: use of strictly standardized median difference with no replicates (SSMD*) in identification of hits

**Fig. 4:**
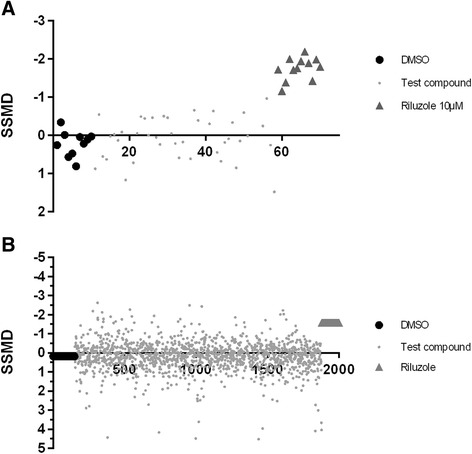
Representative SSMD* scores of a plate and screen replicate from the ZNStress assay. **a** The plate comprising 12 positive controls (riluzole, black dots) and 12 negative control (DMSO, black triangles) and 48 test compounds (grey squares) were calculated for SSMD*. Riluzole shows a clear inhibitory effect and consistently shows a negative SSMD*, while most test compounds and DMSO controls show very little inhibitory activity. **b** 2000 compounds from the MicroSource Spectrum library were tested over 40 plates. Positive control for inhibitor of neuronal stress (riluzole, grey triangle) and negative DMSO controls (black circle) and test compounds (grey circles). A small fraction of drugs in the screen show biological effects comparable to riluzole and a small fraction of compounds in the library also showed positive SSMD values above 2, indicating that they were activators. Compounds that showed similar efficacy in two trials were classified as hits

SSMD* is a method described by Zhang et al in an RNAi screen. Screens that have a single well, with no replicates, require a method to identify hits within a plate. In SSMD* analysis, it is assumed that majority of the compounds in the specific plate tested have no efficacy and thus can be potentially considered as negative controls. A hit in the plate will stand out as an outlier and thus can be identified. Hence the median is used to measure the predominantly inert effects of most compounds in the assay. The advantage of this approach is that the whole plate is treated similarly, and hence only wells that are significantly different from the majority of the wells with no effects are identified. Using this method we tested 48 compounds with 12 positive and 12 negative controls in each plate. The layout of the plate is shown in Additional file 5: Figure S4. Thus, if most of the compounds in the plate have no effect at all, only the wells with riluzole should show up as positive hits in the plate. Figure 4a shows the SSMD scores of a representative plate in the screen. As expected, only the wells with riluzole showed an SSMD* score of < -0.5 or more. This step confirmed that legitimate hits can be identified using this methodology.

#### QC analysis of positive and negative controls within plates and between plates

Using the SSMD* analysis we tested the reproducibility of the assay using riluzole and DMSO controls in each plate in the screen. The screen included 40 plates with 12 positive and 12 negative controls and 48 test compounds in each plate. The whole screen was then repeated a second time. The replicate QC of the screen using positive and negative controls with SSMD* analysis set at a < -0.5 cut-off as hits. The sensitivity of the assay was measured using the formula (#true positive/ (#true positive + #false negative))*100. The specificity of the assay was measured using the formula (#true negative/ (#true negative + #false positive))*100. The assay sensitivity ranged from 94.8–96.8 % and the specificity from 85.2–93.2 % (Table 1). When the SSMD* cut-off for hits was reduced to < -1.0, the sensitivity ranged from 79–81 %, while specificity increased to 96.5–98 % (Table 1).

**Table 1:** Specificity and sensitivity of the screening assay based upon an SSMD threshold of < -0.5

	Replicate 1	Replicate 2
Sensitivity in percentage	94.8 % ± 9.85	96.6 % ± 8.8
Specificity in percentage	85.2 % ± 13.2	93.2 % ± 9.0

The assay statistics from 40 plates in each replicate including 12 positive control (riluzole) and 12 negative control wells. The false positive and false negative numbers were utilized to calculate the specificity and sensitivity of the assay expressed as percentage mean and standard deviation

#### Hit analysis

Using the cut-off values of < -0.5, <-1.0 (Inhibitors) or >1.0 (Activators) the hits were identified. Figure 4b shows the SSMD* values of all drugs tested in the screen along with positive and negative controls on either side. In all plates screened, riluzole always showed activity. 38 inhibitors of neuronal stress were identified below–0.5 and only 7 below–1.0, which represented 1.9 and 0.35 % of the compounds tested (Table 2). Activators with SSMD* scores above 1.0 were 20 (1 %) and 142 or 7.1 % of the compounds were toxic, causing death of the embryos (Table 2). all toxic compounds were rescreened again at 1 and 0.1 μM concentrations to detect if any had activity at lower concentrations. However, none of the toxic compounds showed any stress modulating activity at lower concentrations. The hits observed in the screen are realistic, with a small fraction of the total pool showing biological activity and with the positive and negative controls showing clear and reproducible effects. The hit compounds were diverse comprising of ion channel regulators, steroids, anti-bacterial, anti-oxidants and anti-inflammatory compounds, although only one compound showed efficacy to similar levels as riluzole.

**Table 2:** Statistics of assay hits from both replicates of the high-throughput screen

SSMD Threshold	Number of replicated hits	Number screened	Percentage hits
Below -0.5	38	2000	1.9 %
Below -1.0	7	2000	0.35 %
Above 1.0	20	2000	1 %
Caused Death	142	2000	7.1 %

Compounds that showed similar activity in both trials were identified as hits using the SSMD* threshold criteria as above. SSMD* below -0.5 are inhibitors of neuronal stress and those above 1.0 are activators of the neuronal heat-shock stress response

### Efficacy of compounds previously tested in the sod1 mouse model and/or in ALS clinical trials

**Fig. 5:**
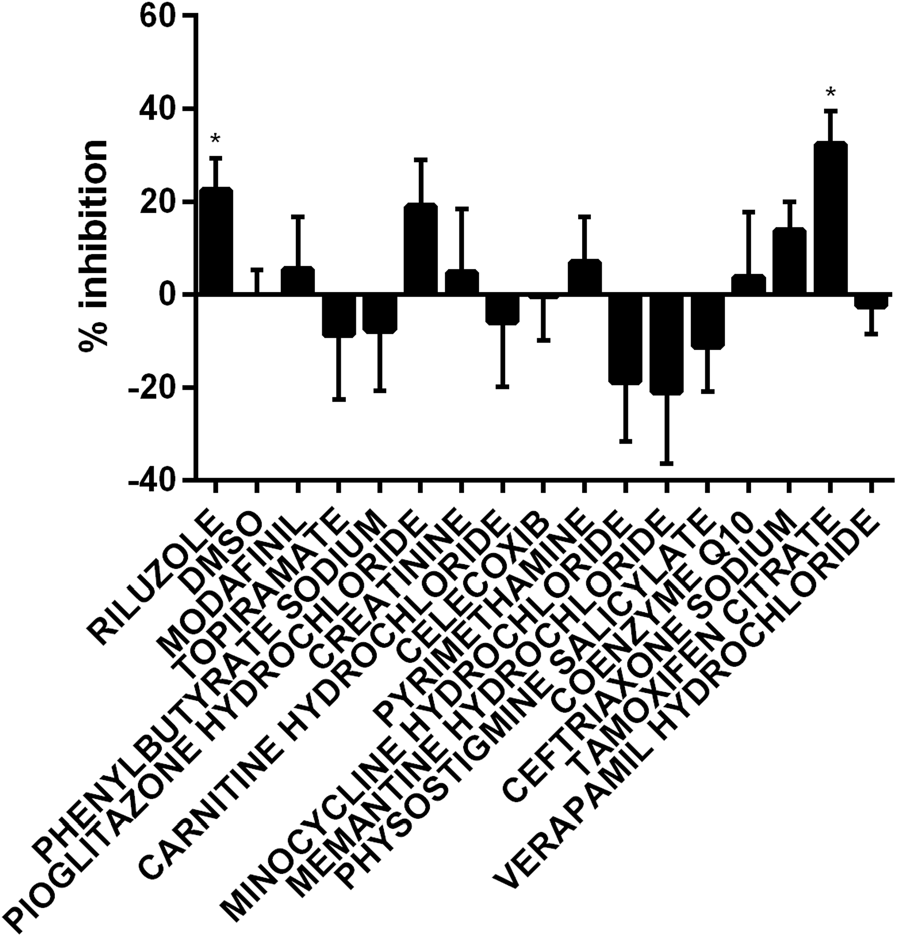
ALS drugs identified in mouse models but lacked clinical efficacy failed to show efficacy in the ZNStress assay. Percentage reduction in DsRed fluorescence of 17 drugs that were tested in the mouse models of ALS from the Microsource Spectrum library. Mean ± SEM

Many drug trials have been performed in the SOD1 mouse model and multiple compounds have shown efficacy [1]. However, many of these compounds when taken to human clinical trials failed to show efficacy. Additionally, several recent publications have cast doubt on the efficacy of compounds previously tested [30–34]. Many of the factors affecting the reproducibility of pre-clinical testing in the SOD1 mouse model have been standardised and new guidelines have been formulated [32]. In order to examine whether the positive drug effects previously identified in the SOD1 mouse model were reproducible in the mutant Sod1 zebrafish model of neuronal stress, we evaluated 17 drugs which included riluzole. Out of all of these compounds only riluzole showed efficacy in reducing neuronal stress (Fig. 5 and Additional file 6: Table S2). These results are in agreement with previously published reports of the lack of efficacy of many of these compounds when tested in follow up pre-clinical studies and/or clinical trials.

#### Dexpramipexole fails to reduce neuronal stress in the mutant sod1 zebrafish model of ALS

**Fig. 6:**
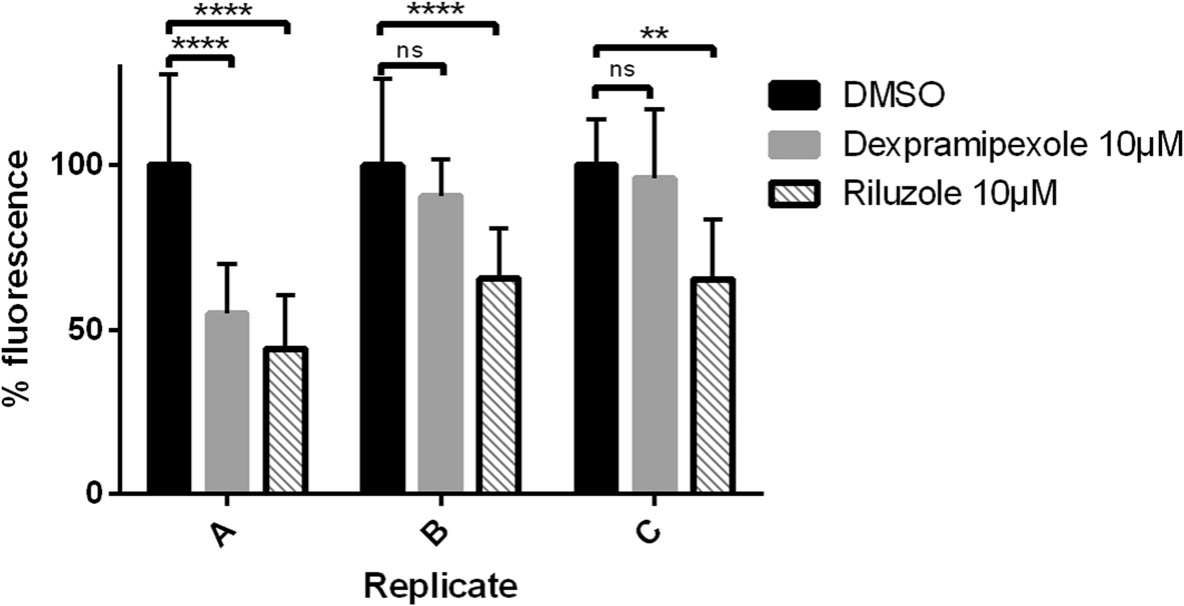
Experimental variability and occurrence of random efficacy readout in drug trials: Dexpramipexole study. In replicate 1 both riluzole and dexpramipexole (both 10 μM) showed a significant reduction in DsRed fluorescence. However in replicate 2 and 3 only riluzole showed a significant reduction in fluorescence. This highlights the need for stringent assay QC and experimental replicates. *N*= >10 for each replicate, error bars show SD

One of the major clinical trials in ALS to fail at phase 3 after promising phase 2 results, was the recent trial of dexpramipexole. Interestingly, prior to the human trial, no pre-clinical mouse data were published. When we tested dexpramipexole using the mutant Sod1 zebrafish model, we observed in the first evaluation a statistically significant effect in the reduction of neuronal stress (Fig. 6a). However, two follow up studies using the same preparation and the same batch of the drug failed to show any reduction in the neuronal stress (Fig. 6b, c). Overall analysis of the pooled data from triplicate experiments showed that dexpramipexole lacked efficacy in reducing neuronal stress in this Zebrafish model system.

## Discussion

The ZNStress assay is a step forward in pre-clinical screening for compounds which may exert neuroprotective effects in ALS, as it links the key characteristics of a transgenic zebrafish line with screening techniques, to deliver a truly high-throughput in vivo drug screen. The only true limiting factor relating to this screen is the number of embryos available each day and the manual pipetting of the zebrafish between plates. In its current format and being run by a single individual, it was comfortably possible to screen 4x96 well plates per day twice per week. This means that a screen of the full library comprising 2000 compounds in duplicate takes 6 weeks to complete. This highlights how suitable zebrafish are for screening and how we are moving towards the ability to screen hundreds of thousands of compounds in vivo to identify potential disease modifiers for human diseases.

Although the primary goal of the reported screen was to identify compounds that reduce neuronal toxicity mediated by mutant Sod1, we were also able to use the same assay to identify compounds that can activate the cellular stress response mediated by up-regulation of the heat-shock protein response, which represents a neuroprotective mechanism [35, 36] (Fig. 1). These compounds act downstream of the initial insult, i.e. the activation of the stress response mediated by mutant Sod1 misfolding and/or its associated toxicity. These compounds are of interest to the ALS field as activation of heat shock proteins has been shown to be neuroprotective and heat shock protein activators, such as arimoclomol, are currently in clinical trials for ALS [35]. Care must be taken as compounds that auto-fluorescence or are toxic also cause an increase in DsRed fluorescence. The compounds that activate the stress response can be further evaluated in secondary screens of efficacy to separate off-target effects from real heat shock protein activation.

The question still remains as to how closely a zebrafish model can mimic a human neurodegenerative disease, especially as this animal model retains the capacity for neuroregeneration. Additionally sod1 mutation is a small sub group comprising of 20 % of familial ALS patients and may not represent the predominant sporadic form of ALS. However, some recent data suggesting wildtype sod1 may be modified to toxic species in a prion like fashion in sporadic ALS [37], warrants the continued utility of sod1 based disease models in ALS research. Nevertheless the wide array of mutations contributing to ALS pathogenesis require additional genetic models to cover a broad spectrum of ALS pathogenesis and warrant the development of a wider net of screening tools. The assay described here is an embryonic/larval screen and hence limits its use to identifying factors involved in early pathogenesis. It is becoming increasingly evident that changes in ALS occur far earlier than clinical manifestation of the disease occur. This poses a challenge in treating the disease. It is also becoming increasingly recognised that potentially clinical stage of disease is too late for therapeutic intervention [19]. Hence, phenotypic identification and early diagnosis are gaining importance in therapeutic development for ALS. There is a more focussed push towards biomarker discovery and therapy development bringing in an era of personalized medicine [38].

The utility of the ZNStress zebrafish model is shown by the efficacy of riluzole, the only approved drug used in the treatment of ALS. Riluzole shows robust inhibition of neuronal stress, demonstrates that drugs which are active in human ALS also show similar activity in a zebrafish model. Interestingly, many of the compounds emerging from murine preclinical studies that failed to show efficacy in human ALS clinical trials, also showed no efficacy in the ZNStress assay (Additional file 5: Table S2). The failure to reproduce positive effects of drugs in the SOD1 mouse model was ascribed to poor quality control in pre-clinical studies, necessitating the development of international guidelines for drug studies in rodent ALS models [39]. Low animal numbers, lack of litter matching, transgene copy number variability and lack of clear endpoints contribute to such spurious effects [39]. The need for a high degree of rigour in any pre-clinical evaluation of efficacy is clearly demonstrated by our experience with dexpramipexole. While one trial with dexpramipexole showed efficacy (Fig. 6a), follow up studies showed no efficacy (Fig. 6b, c). Like all HT screens unknown factors can occasionally lead to a spurious false positive result and hence the hits need to be robustly validated subsequently. Thus, high numbers and multiple screening repeats are key in eliminating false positive results. An important advantage of zebrafish is that we can conduct multiple efficacy studies using hundreds of embryos from multiple clutches, thus greatly enhancing the confidence of drug efficacy before testing in rodent models. We believe that zebrafish models are unlikely to replace mouse screens, but they have the ability to identify lead compounds of interest with high confidence to predict biological activity in a vertebrate system, thus reducing the number of mouse studies required. This approach would be expected to accelerate and reduce the costs of pre-clinical drug discovery.

Future improvement of the screen described here will be to adapt the ZNStress assay readout so that the zebrafish can be kept alive after exposure, allowing genetic modifier and behavioural screens to be performed. An optimal system for this will be the InCell analyser zebrafish plugin as the system can rapidly capture images (96well plate in 2 wavelengths in 10 min at 2x). This will allow a decisive readout on which specific tissues and cell types, show an increase or decrease in fluorescence, alongside the ability to keep the zebrafish alive for downstream evaluation. A further potential improvement to the screen will be the implementation of an automated zebrafish handling system that can selectively genotype and load zebrafish embryos accurately and rapidly into wells, thereby increasing the screen throughput. Unfortunately, these systems are prohibitively expensive currently as they are still in development, but in the future it is likely that these systems will be common place in zebrafish facilities and will further improve the screening throughput.

The ZNStress assay is not limited to this particular zebrafish line, the ALS disease state or the specific field of biology upon which we have focussed in this report. The assay can be used in any fluorescence based zebrafish screen, as it is flexible and can be applied to multiple tissue types. We have developed and utilized the ZNStress assay with transgenic zebrafish carrying *hsp70-DsRed* in the context of *wildtype sod1* to identify neurotoxic genes involved in Parkinson’s disease (unpublished data). In the Parkinson’s disease model, the neuronal stress produced by specific parkinson mutations activate the DsRed reporter in the WT-sod1 transgenic line. The assay process described here utilized imaging systems that can detect fluorescence at any developmental stage. The assay can be shortened or extended for different lengths of drug exposure and has a readout that can be performed at different wavelengths. In future, the development of additional zebrafish models carrying different ALS mutations is necessary for comprehensive therapeutic screening and enable personalized medicine.

## Conclusions

We conclude that HT drug screening using a mutant sod1 zebrafish is a reliable model system which supplemented with secondary assays would be useful in identifying drugs with potential for neuroprotective efficacy in ALS.

## Abbreviations

ALS, amyotrophic lateral sclerosis; DMSO, dimethyl sulfoxide; DsRed, discosoma red fluorescent protein; FDA, food and drug administration; HSP, heat shock protein; HT, high throughput; PDDS, phenotypic drug discovery screen; QC, quality control; SOD, superoxide dismutase; SSMD, strictly standardised median difference; TDDS, target directed drug discovery; UPR, unfolded protein response; ZNStress, zebrafish neuronal stress

## Additional files

Additional file 1: Figure S1. Embryonic quality control. Bright field images from Incell Analyzer 2000 of a representative 2 dpf-pre treatment embryo (Top) and 6 dpf embryos treated with DMSO (Middle) and Riluzole (Bottom). The embryos appear normal and undergo normal development. All embryos utilized in the screen were imaged and quality controlled. (TIF 171 kb) Additional file 2: Table S1. Table of SSMD β-value hit selection criteria. (DOC 32 kb) Additional file 3: Figure S2. Pherastar readout showing the ability to detect different drug effects in the screen. G93Ros10xAb zebrafish dosed with riluzole at 10 μM and DMSO. Zebrafish were scanned in a 30x30 point well scan for DsRed fluorescence. The readout is a spectral representation of DsRed expression from purple/blue = low signal to yellow/red = high signal. (TIF 1147 kb) Additional file 4: Figure S3. Variability in percent inhibition within and between plates in riluzole and DMSO treated wells. A clear separation of effects of riluzole (black) as compared to the DMSO controls (grey) which is close to zero change. The well to well variability and between plate variability as measured by SEM (error bars) shows that variability does not impact the identification of drug effects compared to DMSO controls. (TIF 21 kb) Additional file 5: Figure S4. Plate layout for ZNStress assay. B – Blank, R - Positive control (riluzole), C- Negative control (DMSO), T- Test compound. (TIF 307 kb) Additional file 6: Table S2. Effects of drugs tested in human ALS in the mutant sod1 ZNSstress assay. (DOC 43 kb)
