# Supplementary figures and images for: ZNStress: a high-throughput drug screening protocol for identification of compounds modulating neuronal stress in the transgenic mutant sod1G93R zebrafish model of amyotrophic lateral sclerosis
Source: Mol Neurodegener. 2016 Jul 26;11:56. doi: 10.1186/s13024-016-0122-3 (PMC4962399; doi:10.1186/s13024-016-0122-3)

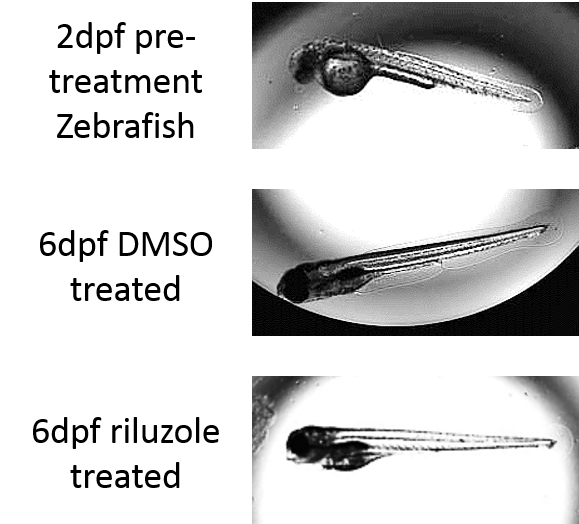

Supplement: Additional file 1: Figure S1. — Embryonic quality control. Bright field images from Incell Analyzer 2000 of a representative 2 dpf-pre treatment embryo (Top) and 6 dpf embryos treated with DMSO (Middle) and Riluzole (Bottom). The embryos appear normal and undergo normal development. All embryos utilized in the screen were imaged and quality controlled. (TIF 171 kb) [file 13024_2016_122_MOESM1_ESM.tif]

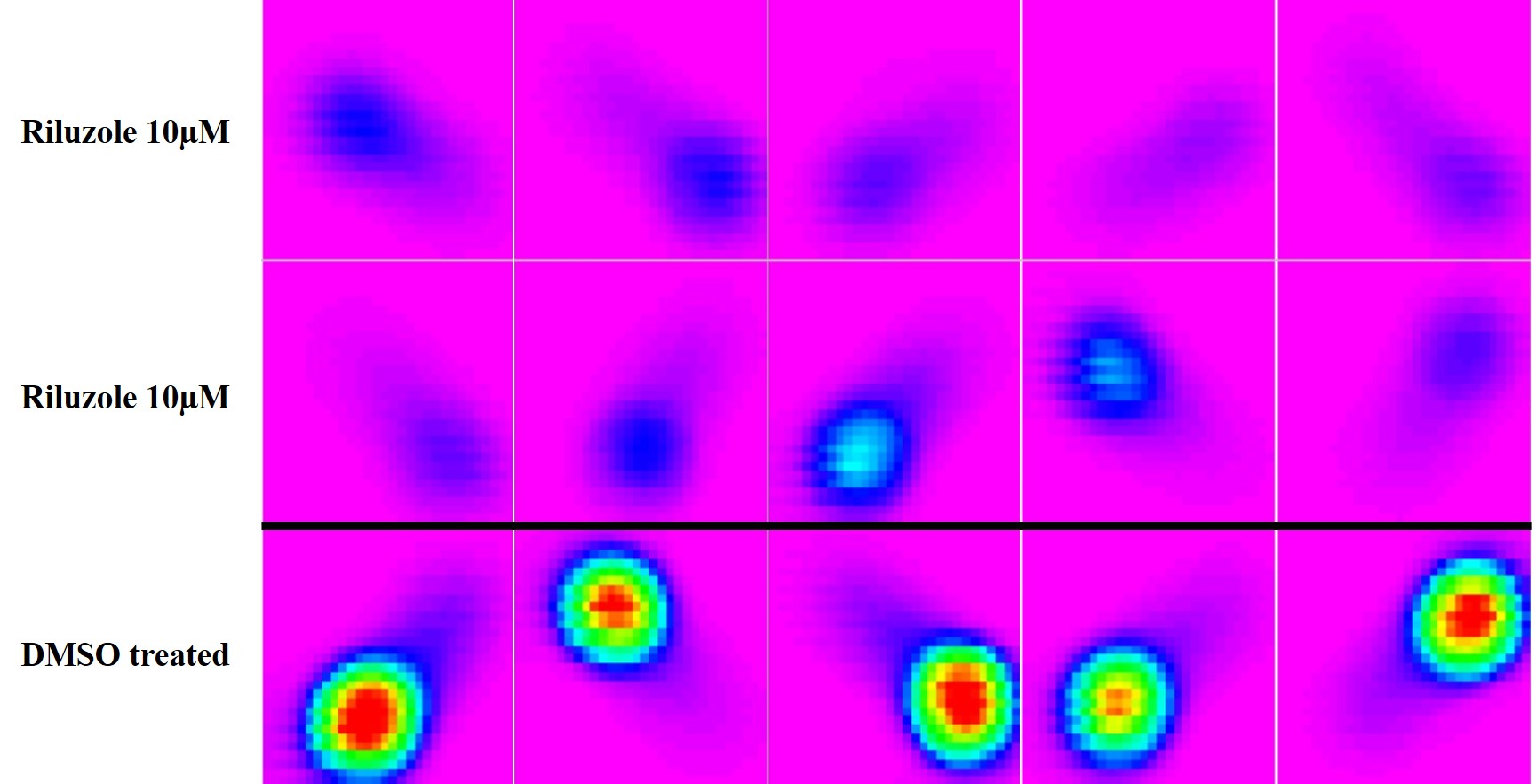

Supplement: Additional file 3: Figure S2. — Pherastar readout showing the ability to detect different drug effects in the screen. G93Ros10xAb zebrafish dosed with riluzole at 10 μM and DMSO. Zebrafish were scanned in a 30x30 point well scan for DsRed fluorescence. The readout is a spectral representation of DsRed expression from purple/blue = low signal to yellow/red = high signal. (TIF 1147 kb) [file 13024_2016_122_MOESM3_ESM.tif]

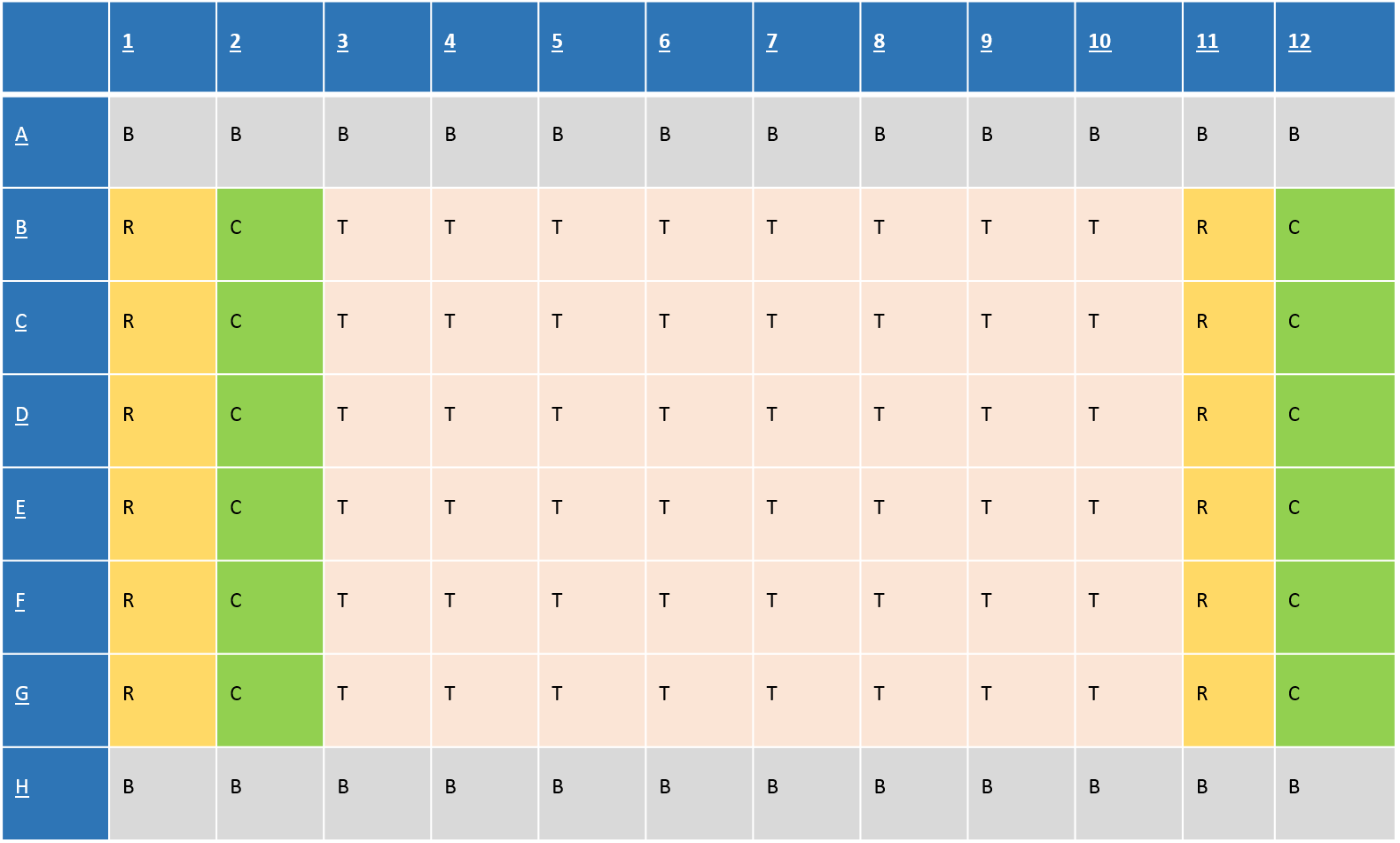

Supplement: Additional file 5: Figure S4. — Plate layout for ZNStress assay. B – Blank, R - Positive control (riluzole), C- Negative control (DMSO), T- Test compound. (TIF 307 kb) [file 13024_2016_122_MOESM5_ESM.tif]
