# Supplementary material for: ZNStress: a high-throughput drug screening protocol for identification of compounds modulating neuronal stress in the transgenic mutant sod1G93R zebrafish model of amyotrophic lateral sclerosis
Source: Mol Neurodegener. 2016 Jul 26;11:56. doi: 10.1186/s13024-016-0122-3 (PMC4962399; doi:10.1186/s13024-016-0122-3)
Supplement: Additional file 2: Table S1. — Table of SSMD β-value hit selection criteria. (DOC 32 kb) [file 13024_2016_122_MOESM2_ESM.doc]

Additional file 2: Table S1: Table of SSMD β-value hit selection criteria.

| Effect subtype | Thresholds for negative SSMD | Thresholds for positive SSMD |
| --- | --- | --- |
| Extremely strong | β < -5 | β > 5 |
| Very strong | − 5 < β < − 3 | 5 > β > 3 |
| Strong | − 3 < β < − 2 | 3 > β > 2 |
| Fairly strong | − 2< β < − 1.645 | 2 > β > 1.645 |
| Moderate | − 1.645 < β < − 1.28 | 1.645 > β > 1.28 |
| Fairly moderate | − 1.28 < β < − 1 | 1.28 > β > 1 |
| Fairly weak | − 1 < β < − 0.75 | 1 > β > 0.75 |
| Weak | − 0.75 < β < − 0.5 | 0.75 > β > 0.5 |
| Very weak | − 0.5 < β < − 0.25 | 0.5 > β > 0.25 |
| Extremely weak | − 0.25 < β < 0 | 0.25 > β > 0 |
| No effect | β = 0 |  |

Note that for this assay a negative SSMD shows a reduction in fluoresence, whereas a positive SSMD indicates an increase in fluoresence.
