# Supplementary material for: ZNStress: a high-throughput drug screening protocol for identification of compounds modulating neuronal stress in the transgenic mutant sod1G93R zebrafish model of amyotrophic lateral sclerosis
Source: Mol Neurodegener. 2016 Jul 26;11:56. doi: 10.1186/s13024-016-0122-3 (PMC4962399; doi:10.1186/s13024-016-0122-3)
Supplement: Additional file 6: Table S2. — Effects of drugs tested in human ALS in the mutant sod1 ZNSstress assay. (DOC 43 kb) [file 13024_2016_122_MOESM6_ESM.doc]

Additional file 6: Table S2: Effects of drugs tested in human ALS in the mutant sod1 ZNSstress assay

| **DRUGS TESTED** | **ZF neuronal stress reduction** | **Potential MOA** | **ALS mouse model** | **Human safety** | **Benefits in ALS** |
| --- | --- | --- | --- | --- | --- |
| RILUZOLE | +++ | Excitotoxicity | + | ++++ | Clinically approved for ALS |
| MODAFINIL | - | Reduce muscle fatigue | Not tested | +++ | Not disease modifying |
| TOPIRAMATE | - | Excitotoxicity | - | + | Negative in a large clinical trial |
| PHENYLBUTYRATE SODIUM | - | HDAC inhibitor | + | +++ | Not tested |
| PIOGLITAZONE HYDROCHLORIDE | Toxic | Inflammation | + | +++ | Pilot study negative |
| CREATINE | - | Mitochondrial respiration | + | +++ | Negative (Cochran review) |
| CARNITINE HYDROCHLORIDE | Toxic | Mitochondrial injury | + | ++ | Pilot study positive |
| CELECOXIB | - | Inflammation | + | +++ | Negative in a large clinical trial |
| PYRIMETHAMINE | - | Reduce sod1 | - | + | Not tested (reduced leukocyte sod1 levels) |
| MINOCYCLINE HYDROCHLORIDE | - | Inflammation | + | +++ | Negative in a large clinical trial |
| MEMANTINE HYDROCHLORIDE | - | Excitotoxicity | + | + | Pilot study negative |
| PHYSOSTIGMINE SALICYLATE | - | Cholineesterase inhibitor | Not tested | ++ | Pilot study negative |
| COENZYME Q10 | - | Mitochondrial respiration | + | ++ | Phase II negative |
| CEFTRIAXONE SODIUM | - | Glutamate transporter | + | +++ | Negative in a large clinical trial |
| TAMOXIFEN CITRATE | Toxic | Neuronal survival | (In viral induced model)+ | ++ | Unpublished pilot study negative |
| VERAPAMIL HYDROCHLORIDE | - | Excitotoxicity | Not tested | +++ | Pilot study negative |
